# Reduction of Circulating Endothelial Cells in Peripheral Blood of ALS Patients

**DOI:** 10.1371/journal.pone.0010614

**Published:** 2010-05-12

**Authors:** Svitlana Garbuzova-Davis, Robert L. Woods, Michael K. Louis, Theresa A. Zesiewicz, Nicole Kuzmin-Nichols, Kelly L. Sullivan, Amber M. Miller, Diana G. Hernandez-Ontiveros, Paul R. Sanberg

**Affiliations:** 1 Center of Excellence for Aging and Brain Repair, College of Medicine, University of South Florida, Tampa, Florida, United States of America; 2 Department of Neurosurgery and Brain Repair, College of Medicine, University of South Florida, Tampa, Florida, United States of America; 3 Department of Neurology, College of Medicine, University of South Florida, Tampa, Florida, United States of America; 4 Department of Molecular Pharmacology and Physiology, College of Medicine, University of South Florida, Tampa, Florida, United States of America; 5 Department of Pathology and Cell Biology, College of Medicine, University of South Florida, Tampa, Florida, United States of America; 6 Department of Psychiatry, College of Medicine, University of South Florida, Tampa, Florida, United States of America; 7 Saneron CCEL Therapeutics, Inc., Tampa, Florida, United States of America; Universidade Federal do Rio de Janeiro (UFRJ), Brazil

## Abstract

**Background:**

Amyotrophic Lateral Sclerosis (ALS) treatment is complicated by the various mechanisms underlying motor neuron degeneration. Recent studies showed that the blood-brain barrier (BBB) and blood-spinal cord barrier (BSCB) are compromised in an animal model of ALS due to endothelial cell degeneration. A later study demonstrated a loss of endothelium integrity in the spinal cords of ALS patients. Since circulating endothelial cells (CECs) in the peripheral blood are associated with endothelium damage, being detached dysfunctional endothelial cells, we hypothesized that CEC levels may reflect endothelium condition in ALS patients.

**Methodology/Principal Findings:**

CEC levels were estimated in whole blood smears from ALS patients with moderate stage (_M_ALS), severe stage (_S_ALS), and healthy controls by CD146 expression using immunocytochemistry. A significant reduction of CECs was detected in _M_ALS and _S_ALS patients.

**Conclusions/Significance:**

CECs did not predict endothelium state in ALS patients; however, endothelial damage and/or impaired endothelium repair may occur in ALS leading to BBB/BSCB dysfunction. Reduced CECs in peripheral blood of ALS patients may indicate different mechanisms of endothelial damage and repair, rather than only detachment of dysfunctional endothelial cells. Although a potential mechanism of CEC reduction is discussed, establishing a reliable indicator of endothelial dysfunction/damage is important for evaluation of BBB/BSCB status in ALS patients during disease progression.

## Introduction

Amyotrophic Lateral Sclerosis (ALS) is a fatal neurodegenerative disorder characterized by motor neuron degeneration in the brain and spinal cord clinically manifesting as progressive muscular weakness leading to paralysis and death. Most ALS cases are sporadic (SALS) with only 5–10% of cases genetically linked (FALS). Of familial cases, 20% show missense mutations in the Cu/Zn superoxide dismutase (SOD1) gene [1]. Numerous hypotheses have been proposed concerning ALS pathogenesis [2]–[7], yet limited therapeutic options exist.

Development of an effective treatment for ALS is complicated by the various underlying disease mechanisms and by the diffuse nature of motor neuron death. One possible mechanism involved in ALS pathogenesis is impairment of the blood-brain barrier (BBB) and blood-spinal cord barrier (BSCB), aggravating motor neuron damage. Recent findings indicated that these barriers are compromised in an animal model of ALS. Originally, we showed structural and functional impairment of BBB/BSCB in G93A SOD1 mice at both early and late disease stages [8], [9]. Evans Blue leakage, downregulation of Glut-1, and laminin expression were detected in spinal cord microvessels. Importantly, capillary ultrastructure revealed endothelial cell (EC) degeneration, which, along with astrocyte degeneration, compromised the BBB/BSCB, resulting in vascular leakage. These initial findings were extended, showing endothelial damage in SOD1 mutants with different biochemical characteristics [10]. Importantly, Zhong et al. [10] revealed for the first time that SOD1 mutant-mediated endothelial damage leads to BSCB breakdown prior to motor neuron degeneration and neurovascular inflammatory response, indicating that this damage “was a central contributor to disease initiation”. Reduced levels of tight junction proteins ZO-1, occludin, and claudin-5 were also first demonstrated before disease onset. Moreover, primary findings on significant (30–45%) reduction in blood flow through the cervical and lumbar spinal cord in pre-symptomatic G93A SOD1 mice [10] may lead to vascular hypoperfusion and accelerate motor neuron degeneration. Although reduced capillary blood flow recently was shown in brains of ALS patients in correlation with disease severity [11], a link between this reduction and BBB/BSCB dysfunction still needs to be established. BBB/BSCB impairment was also found in SOD1 rats [12], [13]. Edema-linked BBB/BSCB openings and water transport abnormalities were also noted. Additionally, microhemorrhages and hemosiderin deposits were found in spinal cord parenchyma of both mouse [10] and rat [13] models of ALS demonstrating BSCB openings. A more recent study [14] demonstrated lost endothelium integrity by decreased mRNA transcription of tight junction proteins in autopsied human spinal cords from both sporadic and familial forms of ALS, strengthening the likelihood that BSCB disruption contributes to disease progression.

Based on recent findings of CNS microvascular pathology in ALS including compromised BBB/BSCB in both patients and animal models, ALS can now be considered a neurovascular disease. Neurovascular dysfunction has been shown to significantly contribute to the pathogenesis of Alzheimer's disease (AD) [15], [16], stroke [17], [18], and multiple sclerosis [19], [20]. In AD, for example, endothelial damage through downregulation of endothelial MEOX2 homeobox gene, a regulator of vascular differentiation, may lead to impaired brain angiogenesis, vessel malformation and regression, reduced capillary density and cerebral blood flow, and BBB pathology [15]. Moreover, neurovascular and BBB mechanisms may importantly contribution to both onset and progression of AD [16].

Newly discovered EC damage in ALS, preceding entry of harmful blood-borne substances into areas of motor neuron degeneration, may have implications for disease pathogenesis [21]. Endothelium dysfunction in ALS may be due to impaired endothelialization. Circulating endothelial cell (CEC) levels in peripheral blood might correspond to endothelial damage or dysfunction [22], [23]. Increased numbers of CECs have been detected in peripheral blood of patients with vascular diseases such as myocardial infarction [24], [25] and acute ischemic stroke [26] correlating with plasma markers of endothelial dysfunction. We hypothesized that elevated CEC levels reflect deterioration of the endothelial lining in ALS as a result of BBB/BSCB breakdown. The aim of this study was to determine the levels of CECs in the peripheral blood of ALS patients at different stages of disease.

## Results

### ALS patients and healthy controls

Circulating endothelial cell (CEC) levels in peripheral blood from thirteen ALS patients and six healthy controls were determined using immunocytochemical analysis. Patients had diagnoses of ALS for 23.2±4.2 months (range 7–53 months) with ALSFRS-R scores of 30.1±2.0 (range 21–41) (Table 1). Sporadic ALS (SALS) was indicated in eleven patients and two patients had a familial history of ALS (FALS). At clinic visit, ALS patients had moderate (_M_ALS, n = 6, ALSFRS-R scores of 36.4±2.6, range 30–41) or severe (_S_ALS, n = 7, ALSFRS-R scores of 24.6±0.97, range 21–27) disease. Each healthy control scored 48. Both FALS patients had severe stage disease. ALS patients were taking various anti-inflammatory medications and a standard dose of riluzole. Healthy controls were medication-free at least six months prior to blood collection. Patients had ALS onset at upper, lower or both limbs. No patients had bulbar onset. All study participants tested negative for infectious diseases: HIV, hepatitis B and C, syphilis, and HTLV I&II.

**Table 1:** ALS Patient and Healthy Control Demographics.

Subject	n	Sex (M/F)	ALSFRS-R Score (mean ± S.E.M.)	Age (years, mean ± S.E.M.)	Disease Duration from Diagnosis (months, mean ± S.E.M.)
All ALS Patients	13	12/1	30.08±2.04	53.62±2.64	23.15±4.20
			*Range:* 21–41	*Range:* 39–69	*Range:* 7–53
ALS Patients with Moderate Stage (_M_ALS)	6	5/1	36.40±2.56	55.67±2.70	20.00±5.28
			*Range:* 30–41	*Range:* 45–63	*Range:* 7–32
ALS Patients with Severe Stage (_S_ALS)	7	7/0	24.57±0.97	51.90±4.63	25.86±6.94
			*Range:* 21–27	*Range:* 39–69	*Range:* 7–53
Healthy Control Subjects	6	3/3	48±0	61.33±5.28	N/A
			N/A	*Range:* 38–69	N/A

### Circulating endothelial cells in peripheral blood

**Figure 1:**
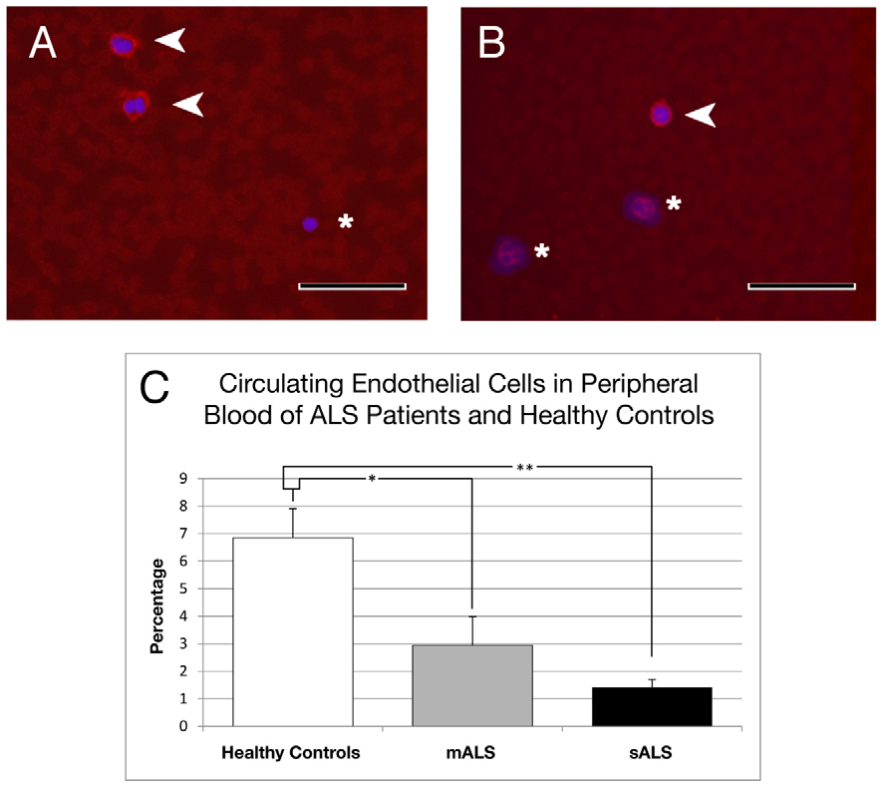
Immunocytochemical analysis of CD146^+^ cells in whole blood smears. (**A**) A few positive CD146 cells (red, arrows) were found in blood smears of control subjects and (**B**) even fewer in _M_ALS or _S_ALS (red, arrow) patients. Asterisks indicate CD146^−^ cells in A and B. Images are merged with DAPI counterlabeled nuclei (blue). Scale bar in A and B is 50 µm. (**C**) Percentages of CD146^+^ cells significantly decreased in _M_ALS and _S_ALS patients compared to healthy controls. *p<0.05, **p<0.01.

Immunocytochemical analysis of CD146^+^ cells in peripheral blood smears demonstrated significantly decreased CECs in _M_ALS patients (2.94±1.09%, *p*<0.05) and _S_ALS patients (1.41±0.29%, *p*<0.01) vs. controls (6.85±1.06%) (Figure 1C). These results were confirmed by microscopic observation of CD146^+^ cells in blood smears; few positive CECs were found in _S_ALS or _M_ALS patients (Figure 1A and B). Interestingly, one FALS patient (sALS group, ALSFRS-R score 24) had the lowest numbers of CD146^+^ (0.85%) cells of any patient.

## Discussion

Integrity of the endothelial cell layer is normally maintained by continuous renewal with a low basal replication rate of 0–1% per day [22], [27]. However, pathological conditions might trigger endothelial cell response. Increased numbers of CECs, considered detached dysfunctional endothelial cells, are associated with endothelial damage in various vascular diseases and correlate with plasma markers (i.e. von Willebrand factor, soluble E-selectin) of endothelial dysfunction [22]–[26], [28], [29]. Recently, CECs have been proposed as a specific biomarker of endothelial damage [22], [23] and even as a predictor of major cardiovascular endpoints [30]. CECs differ from endothelial progenitor cells (EPCs) by their origin; CECs are derived from mature endothelium and EPCs originate from bone marrow. The EPCs circulate in the blood [31] and can migrate to sites of endothelial injury, incorporate into the endothelium and thereby repair damage [32]. Thus, increased numbers of CECs reflect endothelium damage while increased EPCs indicate repair processes [23], [27], [33]. However, impaired or insufficient mobilization of circulating bone marrow derived endothelial progenitor cells, “as a cellular reservoir to replace dysfunctional endothelium”, might contribute to dysfunction of endothelium and further disease progression [24].

The present study examined CEC levels in the peripheral blood smears from ALS patients at different disease stages. CECs in ALS patients at moderate or severe stages of disease and age matched healthy controls were evaluated by immunocytochemistry using CD146 marker, a 130 kDa membrane glycoprotein. Analysis of CECs showed a significant decrease of CD146^+^ cells in patients with either moderate or severe course of disease. These results conflict with the hypothesis of detaching dysfunctional endothelial cells [23], [33]. In healthy adults, CECs are rarely detected [22], [27]. More than 2 fold and 6 fold decreased CECs found in _M_ALS and _S_ALS patients, respectively, from control subject levels might indicate different mechanisms of endothelial damage and/or repair. Possibly, dysfunctional or damaged endothelial cells did not detach from the endothelium and remained within the endothelial layer. “Healthy” cells, presumably differentiated EPCs, may be attaching atop damaged cells, forming a multilayer endothelium. We base this suggestion on observations from a previous G93A mouse study [8], noting numerous capillaries surrounded by multiple layers of endothelial cells and basement membrane in the brainstem and spinal cords of these mice compared to the monolayer endothelium found in control mice. The outer layer of endothelium in symptomatic G93A mice contained swollen endothelial cells with severely vacuolated cytoplasm and intracellular edema. In our ongoing study (unpublished data), ultrastructural examination of BBB/BSCB condition in the post-mortem brainstem and spinal cord of ALS patients showed multiple endothelial layers in some microvessels. Possibly, degenerate abluminal endothelial cells are removed by perivascular macrophages. Although needing confirmation, this could explain the limited appearance of CECs in blood of ALS patients.

Since ALS is a progressive disorder, further endothelial cell alterations could appear during the disease course. Damage of endothelial cells can be due to cell activation by pro-inflammatory cytokines, various growth factors, infectious agents, or oxidative stress. Sufficient evidence exists of inflammatory reactions in ALS. In the brain and spinal cord of both ALS patient and animal models, large numbers of activated microglia and astrocytes, along with inflammatory cell infiltration, have been observed [34]–[41]. These inflammatory effectors can secrete numerous inflammatory cytokines. Increased macrophage-secreted cytokines such as interleukin (IL) 1α, IL 1β and IL 1RA [42], cyclooxygenase type 2 (Cox-2) enzyme [34], [43], [44], and up-regulation of the tumor necrosis factor-alpha (TNF-α) gene [42], [45], and inducible nitric oxide syntheses [46] make these pro-inflammatory cytokines and enzymes likely candidates for the effectors damaging not only motor neurons but also endothelial cells. Moreover, IgG has been noted in ventral horn endothelial cells of post-mortem spinal cord from ALS patients [47]. Additionally, SOD1 mutations might further endothelial cell damage in FALS by increasing oxidative stress. Thus, prolonged inflammation in ALS could induce endothelium impairment leading to BBB/BSCB breakdown. Therefore, evaluation of plasma markers of endothelial dysfunction or damage such as von Willebrand factor and soluble E-selectin might be important.

Additionally, molecular mechanisms of vascular dysfunction and vascular contributions to motor neuron degeneration in ALS should receive special attention. Possibly those specific factor alterations may affect endothelial cell damage. It has been shown, for example, that overexpression of the two transcription factors, serum response factor and myocardin, in the cerebral vascular smooth muscle cells in AD, mediated arterial hypercontractility leading to reduced cerebral blood flow [48] and facilitated cerebral amyloid angiopathy via accumulation of the cytotoxic amyloid beta-peptide in cerebral vessels [49] leading to disease progression.

Alternatively, the mechanism of the endothelial cell layer repair might be impaired in ALS due to insufficient numbers of EPCs mobilized from bone marrow. Although established markers for EPC levels in blood were not examined, it has been showed that CD146^+^ circulating cells, beside CECs, contain a subpopulation of EPCs [50]. Hence, decreased CECs in peripheral blood of ALS patients might indicate inadequate EPC numbers which impair endothelial cell replacement and thereby compromise integrity of the BBB/BSCB in ALS. If this is an issue, approaches for enhancing capillary integrity in the CNS should be developed. One possible approach is cell therapy with the goal of replacing ECs and/or stimulating production of EPCs. Another possibility is protecting the endothelial cell lining by direct cytoprotection or by eliminating microenvironment influences. Promising results have been achieved after intraperitoneal administration of activated protein C (APC) or APC analogs, which have anticoagulant and cytoprotective activities, into G93A SOD1 mice after disease onset. This administration retarded disease progression and increase lifespan by extension duration of the symptomatic phase [51]. These beneficial APC effects were attended by transcriptional downregulation of mutant SOD1 in motor neurons, microglia, and cells comprising microvessels, but not mediated by SOD1 reduction within endothelial cells. It is possible that a more neuroprotective effect can be attained by APC administration into systemic circulation since it crosses the BSCB via endothelial protein C receptor.

In summary, our results suggest that endothelial damage and impaired endothelium repair occur in ALS leading to BBB/BSCB impairment. However, CECs did not reflect endothelial state in ALS. It is possible that mechanisms of endothelial damage and repair, other than detachment of dysfunctional endothelial cells, occur in ALS. Establishing additional indicators of endothelial dysfunction or damage is important for the evaluation of BBB/BSCB status in ALS patients during disease progression. We believe that results of the present study might aid researchers and clinicians in understanding disease-related endothelium damage and in developing effective treatments for ALS.

## Materials and Methods

### Ethics Statement

Patients gave informed written consent prior to inclusion in the study. This study was approved by the Ethics Committees/Institutional Review Board of the University of South Florida (USF, IRB #103861). The study was conducted according to International standards of Good Clinical Practice-(ICH guidelines and the Helsinki Declaration).

### Subjects

Thirteen clinically definite ALS patients (12 males and 1 female, mean age 53.6±2.6 years) and six healthy controls (3 males and 3 females, mean age 61.3±5.3 years) entered our study, visiting the University of South Florida clinic (Table 1). All patients and controls were Caucasian. Diagnoses of ALS were previously established according to the EI Escorial Word Federation of Neurology criteria [52], [53] at various ALS clinics/centers. The Revised ALS Functional Rating Scale (ALSFRS-R), scored from 0 to 48, was used to evaluate overall patient functional status [54]. The ALSFRS-R score was updated for each patient when blood was drawn. Control subjects had no neurological, immunological or psychiatric disease. Each participant in the study signed an informed consent form prior to enrolling.

### Collection of peripheral blood samples

The peripheral blood samples (∼90 ml) were obtained by venipuncture from each patient and healthy control in accordance with study protocols. Blood was drawn and collected into a sterile 10 ml tube (BD Vacutainer, REF 366643) containing K2 EDTA (K2E) and a sterile 10 ml tube with a silicone coated interior (BD Vacutainer, Serum, REF 367820) containing a clot activator. A portion of each collected blood sample was sent to the Oklahoma Blood Institute (Oklahoma, OK) for infectious disease (HIV, hepatitis B and C, syphilis, CMV, and HTLV I&II) testing. Another portion of the sample was used for blood smears in this study while the remaining blood is being stored for use in later additional studies.

Blood smears from each blood sample were fixed in methanol (Fisher Scientific) for 5 min for immunocytochemical analysis of CECs. Blood smears were stored at −20°C.

### Immunocytochemical analysis of CECs in peripheral blood

The blood smears were used for immunocytochemical analysis of CECs by CD146 antibody. Blood smears were rinsed three times for 10 min each with 0.1 M PBS (pH 7.2). The mouse monoclonal anti-human antibody CD146 (1∶200, Chemicon) was applied to each slide after 60 min pre-incubation with 10% normal goat serum (Vector) and Triton X100 in PBS. After incubating overnight at 4°C, slides were washed three times in PBS and incubated with goat anti-mouse secondary antibody conjugated to rhodamine (1∶1200, Alexa Flour 594, Invitrogen) for 2 hrs at room temperature. The slides were then rinsed in PBS, coverslipped with Vectashield (DAPI, Vector) and examined under an epifluorescence Olympus BX60 microscope. The CD146^+^ cells were counted in the entire slide. The percentage of CD146^+^ cells was determined from the total number of DAPI stained MNC cells.

### Statistical Analyses

Data are presented as means ± S.E.M. Data showed normal distribution and were analyzed by a one-way ANOVA with Tukey-Kramer Multiple Comparison post-hoc test. GraphPad InStat (*GraphPad Software, Inc.*) software was used.

## References

[1] Rosen DR, Siddique T, Patterson D, Figlewicz DA, Sapp P (1993). Mutations in Cu/Zn superoxide dismutase gene are associated with familial amyotrophic lateral sclerosis.. Nature.

[2] Cleveland DW, Rothstein JD (2001). From Charcot to Lou Gehrig: deciphering selective motor neuron death in ALS.. Nat Rev Neurosci.

[3] Strong MJ, Kesavapany S, Pant HC (2005). The pathobiology of amyotrophic lateral sclerosis: a proteinopathy?. J Neuropathol Exp Neurol.

[4] Consilvio C, Vincent AM, Feldman EL (2004). Neuroinflammation, COX-2, and ALS–a dual role?. Exp Neurol.

[5] Bruijn LI, Miller TM, Cleveland DW (2004). Unraveling the mechanisms involved in motor neuron degeneration in ALS.. Annu Rev Neurosci.

[6] Pasinelli P, Brown RH (2006). Molecular biology of amyotrophic lateral sclerosis: insights from genetics.. Nat Rev Neurosci.

[7] Mitchell JD, Borasio GD (2007). Amyotrophic lateral sclerosis.. Lancet.

[8] Garbuzova-Davis S, Haller E, Saporta S, Kolomey I, Nicosia SV (2007). Ultrastructure of blood–brain barrier and blood–spinal cord barrier in SOD1 mice modeling ALS.. Brain Res.

[9] Garbuzova-Davis S, Saporta S, Haller E, Kolomey I, Bennett SP (2007). Evidence of compromised blood-spinal cord barrier in early and late symptomatic SOD1 mice modeling ALS.. http://www.plosone.org/article/info%3Adoi%2F10.1371%2Fjournal.pone.0001205.

[10] Zhong Z, Deane R, Ali Z, Parisi M, Shapovalov Y (2008). ALS-causing SOD1 mutants generate vascular changes prior to motor neuron degeneration.. Nat Neurosci.

[11] Rule RR, Schuff N, Miller RG, Weiner MW (2010). Gray matter perfusion correlates with disease severity in ALS.. Neurology.

[12] Nicaise C, Soyfoo MS, Authelet M, De Decker R, Bataveljic D (2009). Aquaporin-4 overexpression in rat ALS model.. Anat Rec (Hoboken).

[13] Nicaise C, Mitrecic D, Demetter P, De Decker R, Authelet M (2009). Impaired blood-brain and blood-spinal cord barriers in mutant SOD1-linked ALS rat.. Brain Res.

[14] Henkel JS, Beers DR, Wen S, Bowser R, Appel SH (2009). Decreased mRNA expression of tight junction proteins in lumbar spinal cords of patients with ALS.. Neurology.

[15] Wu Z, Guo H, Chow N, Sallstrom J, Bell RD (2005). Role of the MEOX2 homeobox gene in neurovascular dysfunction in Alzheimer disease.. Nat Med.

[16] Bell RD, Zlokovic BV (2009). Neurovascular mechanisms and blood-brain barrier disorder in Alzheimer's disease.. Acta Neuropathol.

[17] Avata C, Ropper AH (2002). Ischaemic brain oedema.. J Clin Neurosci.

[18] Jung JE, Kim GS, Chen H, Maier CM, Narasimhan P (2010). Reperfusion and neurovascular dysfunction in stroke: from basic mechanisms to potential strategies for neuroprotection.. Mol Neurobiol Feb 17. [Epub ahead of print].

[19] Kirk J, Plumb J, Mirakhur M, McQuaid S (2003). Tight junctional abnormality in multiple sclerosis white matter affects all calibers of vessel and associated with blood-brain barrier leakage and active demyelination.. J Pathol.

[20] Vos CMP, Geurts JJG, Montagne L, van Haastert ES, Bö L (2005). Blood-brain barrier alterations in both focal and diffuse abnormalities on postmortem MRI in multiple sclerosis.. Neurobiol Dis.

[21] Garbuzova-Davis S, Saporta S, Sanberg PR (2008). Implications of blood-brain barrier disruption in ALS.. Amyotroph Lateral Scler.

[22] Blann AD, Woywodt A, Bertolini F, Bull TM, Buyon JP (2005). Circulating endothelial cells. Biomarker of vascular disease.. Thromb Haemost.

[23] Erdbruegger U, Haubitz M, Woywodt A (2006). Circulating endothelial cells: a novel marker of endothelial damage.. Clin Chim Acta.

[24] Hill JM, Zalos G, Halcox JP, Schenke WH, Waclawiw MA (2003). Circulating endothelial progenitor cells, vascular function, and cardiovascular risk. N Eng.. J Med.

[25] Chong AY, Lip GY, Freestone B, Blann AD (2006). Increased circulating endothelial cells in acute heart failure: comparison with von Willebrand factor and soluble E-selectin.. Eur J Heart Fail.

[26] Nadar SK, Lip GY, Lee KW, Blann AD (2005). Circulating endothelial cells in acute ischaemic stroke.. Thromb Haemost.

[27] Hunting CB, Noort WA, Zwaginga JJ (2005). Circulating endothelial (progenitor) cells reflect the state of the endothelium: vascular injury, repair and neovascularization.. Vox Sang.

[28] Dome B, Timar J, Ladanyi A, Paku S, Renyi-Vamos F (2009). Circulating endothelial cells, bone marrow-derived endothelial progenitor cells and proangiogenic hematopoietic cells in cancer: From biology to therapy.. Crit Rev Oncol Hematol.

[29] Goon PK, Lip GY, Stonelake PS, Blann AD (2009). Circulating endothelial cells and circulating progenitor cells in breast cancer: relationship to endothelial damage/dysfunction/apoptosis, clinicopathologic factors, and the Nottingham Prognostic Index.. Neoplasia.

[30] Lee KW, Lip GY, Tayebjee M, Foster W, Blann AD (2005). Circulating endothelial cells, von Willebrand factor, interleukin-6, and prognosis in patients with acute coronary syndromes.. Blood.

[31] Asahara T, Murohara T, Sullivan A, Silver M, van der Zee R (1997). Isolation of putative progenitor endothelial cells for angiogenesis.. Science.

[32] Rafii S (2000). Circulating endothelial precursors: mystery, reality, and promise.. J Clin Invest.

[33] Woywodt A, Bahlmann FH, De Groot K, Haller H, Haubitz M (2002). Circulating endothelial cells: life, death, detachment and repair of the endothelial cell layer.. Nephrol Dial Transplant.

[34] McGeer PL, McGeer EG (2002). Inflammatory processes in amyotrophic lateral sclerosis.. Muscle Nerve.

[35] Boillée S, Vande Velde C, Cleveland DW (2006). ALS: a disease of motor neurons and their nonneuronal neighbors.. Neuron.

[36] Cho KJ, Chung YH, Shin C, Shin DH, Kim YS (1999). Reactive astrocytes express p53 in the spinal cord of transgenic mice expressing a human Cu/Zn SOD mutation.. Neuroreport.

[37] Henkel JS, Engelhardt JI, Siklós L, Simpson EP, Kim SH (2004). Presence of dendritic cells, MCP-1, and activated microglia/macrophages in amyotrophic lateral sclerosis spinal cord tissue.. Ann Neurol.

[38] Levine JB, Kong J, Nadler M, Xu Z (1999). Astrocytes interact intimately with degenerating motor neurons in mouse amyotrophic lateral sclerosis (ALS).. Glia.

[39] Appel SH, Simpson EP (2001). Activated microglia: the silent executioner in neurodegenerative disease?. Curr Neurol Neurosci Rep.

[40] Engelhardt JI, Tajt J, Appel SH (1993). Lymphocytic infiltrates in the spinal cord in amyotrophic lateral sclerosis.. Arch Neurol.

[41] Alexianu ME, Kozovska M, Appel SH (2001). Immune reactivity in a mouse model of familial ALS correlates with disease progression.. Neurology.

[42] Hensley K, Floyd RA, Gordon B, Mou S, Pye QN (2002). Temporal patterns of cytokine and apoptosis-related gene expression in spinal cords of the G93A-SOD1 mouse model of amyotrophic lateral sclerosis.. J Neurochem.

[43] Almer G, Guégan C, Teismann P, Naini A, Rosoklija G (2001). Increased expression of the pro-inflammatory enzyme cyclooxygenase-2 in amyotrophic lateral sclerosis.. Ann Neurol.

[44] Graves MC, Fiala M, Dinglasan LA, Liu NQ, Sayre J (2004). Inflammation in amyotrophic lateral sclerosis spinal cord and brain is mediated by activated macrophages, mast cells and T cells.. Amyotroph Lateral Scler Other Motor Neuron Disord.

[45] Elliott JL (2001). Cytokine upregulation in a murine model of familial amyotrophic lateral sclerosis.. Brain Res Mol Brain Res.

[46] Almer G, Vukosavi C S, Romero N, Przedborski S (1999). Inducible nitric oxide synthase up-regulation in a transgenic mouse model of familial amyotrophic lateral sclerosis.. J Neurochem.

[47] Engelhardt JI, Soós J, Obál I, Vigh L, Siklós L (2005). Subcellular localization of IgG from sera of ALS patients in the nervous system.. Acta Neurol Scand.

[48] Chow N, Bell RD, Deane R, Streb JW, Chen J (2007). Serum response factor and myocardin mediate arterial hypercontractility and cerebral blood flow dysregulation in Alzheimer's phenotype.. Proc Natl Acad Sci U S A.

[49] Bell RD, Deane R, Chow N, Long X, Sagare A (2009). SRF and myocardin regulate LRP-mediated amyloid-beta clearance in brain vascular cells.. Nat Cell Biol.

[50] Delorme B, Basire A, Gentile C, Sabatier F, Monsonis F (2005). Presence of endothelial progenitor cells, distinct from mature endothelial cells, within human CD146+ blood cells.. Thromb Haemost.

[51] Zhong Z, Ilieva H, Hallagan L, Bell R, Singh I (2009). Activated protein C therapy slows ALS-like disease in mice by transcriptionally inhibiting SOD1 in motor neurons and microglia cells.. J Clin Invest.

[52] Brooks BR (1994). El Escorial World Federation of Neurology criteria for the diagnosis of amyotrophic lateral sclerosis. Subcommittee on Motor Neuron Diseases/Amyotrophic Lateral Sclerosis of the World Federation of Neurology Research Group on Neuromuscular Diseases and the El Escorial “Clinical limits of amyotrophic lateral sclerosis” workshop contributors.. J Neurol Sci.

[53] Brooks BR, Miller RG, Swash M, Munsa TL (2000). World Federation of neurology Research Group on Motor Neuron Diseases. El Escorial revisited: revised criteria for the diagnosis of amyotrophic lateral sclerosis.. Amyotroph Lateral Scler Other Motor Neuron Disord.

[54] Cedarbaum JM, Stambler N, Malta E, Fuller C, Hilt D (1999). The ALSFRS-R: a revised ALS functional rating scale that incorporates assessments of respiratory function. BDNF ALS Study Group (Phase III).. J Neurol Sci.

